# Salivary Gland Carcinoma: Novel Targets to Overcome Treatment Resistance in Advanced Disease

**DOI:** 10.3389/fonc.2020.580141

**Published:** 2020-10-22

**Authors:** Larissa Di Villeneuve, Ive Lima Souza, Fernanda Davila Sampaio Tolentino, Renata Ferrarotto, Gustavo Schvartsman

**Affiliations:** ^1^Department of Medical Oncology, Hospital Israelita Albert Einstein, São Paulo, Brazil; ^2^Department of Head and Neck Medical Oncology, The University of Texas MD Anderson Cancer Center, Houston, TX, United States

**Keywords:** salivary gland cancer, molecular targeted therapy, androgen receptor, immunotherapy, ERBB-2 receptor, gene fusion, drug resistance

## Abstract

Salivary gland carcinomas (SGC) account for less than 5% of head and neck malignant neoplasms, further subcategorized in over 20 histological subtypes. For the most part, treatment for advanced disease is guided by morphology. SGC in general respond poorly to standard chemotherapy, with short durability and significant toxicity. More recently, next-generation sequencing provided significant input on the molecular characterization of each SGC subtype, not only improving diagnostic differentiation between morphologically similar tumor types, but also identifying novel driver pathways that determine tumor biology and may be amenable to targeted therapy. Amongst the most common histological subtype is adenoid cystic carcinoma, which often harbors a chromosome translocation resulting in a *MYB-NFIB* oncogene, with various degrees of Myb expression. In a smaller subset, *NOTCH1* mutations occur, conferring a more aggressive disease and potential sensitivity to Notch inhibitors. Salivary duct carcinomas may overexpress Her-2 and androgen receptor, with promising clinical outcomes after exposure to targeted therapies approved for other indications. Secretory carcinoma, previously known as mammary analogue secretory carcinoma, is distinguished by an *ETV6-NTRK3* fusion that can both help differentiate it from its morphologically similar acinar cell carcinoma and also make it susceptible to Trk inhibitors. In the present article, we discuss the molecular abnormalities, their impact on tumor biology, and therapeutic opportunities for the most common SGC subtypes and review published and ongoing clinical trials and future perspectives for this rare diseases.

## Introduction

Salivary gland carcinoma (SGC) is a rare tumor and represents ~6% of head and neck cancers (1). Malignant tumors of the salivary glands constitute a heterogeneous group of neoplasms that vary depending on the histology and their anatomical location. According to the 2017 WHO Classification, there are 24 malignant histological subtypes (2). The most prevalent are mucoepidermoid carcinoma (MEC), representing around a third of SGC cases, followed by adenoid cystic carcinoma (ACC), with 23.8% (3). The parotid gland is the most frequent site of salivary gland tumors, although only 25% of such lesions are malignant. SGC can also originate in the submandibular glands (40–45% of the tumors are malignant), sublingual glands (70–90% are malignant), and minor salivary glands (50% are malignant) (4).

Treatment for metastatic disease is still mostly based on chemotherapy, despite low response, and survival rates (5). Currently, encouraging progress in immunohistochemical and molecular alterations, such as the presence of an *NTRK* fusion, overexpression of Her-2 and androgen receptor, has been made to improve outcomes with targeted therapy.

The aim of this article is to review the main molecular and immunohistochemical characteristics of the most common histological subtypes of SGC, in addition to reviewing current data on biomarker-driven targeted therapy and genomic findings that may be potentially actionable in the future.

## Mucoepidermoid Carcinoma

MEC is the most common SGC (6). In addition to clinical staging, the grade of the tumor is also a prognostic factor in MEC and may guide treatment decision (7). Despite its prevalence, it is one of the subtypes with the least breakthroughs achieved so far.

A unique t (8, 9) translocation, leading to the *CRTC1/MAML2* fusion, is present in 56–82% of all MECs (10, 11). This fusion protein induces aberrant activation of the Notch signaling pathway, inducing cell proliferation and, therefore, tumor progression (12). Data on how this abnormality impacts tumor biology and prognosis are conflicting. While some series indicate that fusion-positive MECs were diagnosed at an earlier stage, with lower grade, and a better prognosis (8, 12, 13), other studies do not suggest a prognostic role for the translocation (10, 14). *CRTC1-MAML2*-positive cells were sensitive to epidermal growth factor receptor (*EGFR*) tyrosine kinase inhibition pre-clinically, suggesting a potential role for such drugs (15).

The most common genomic abnormalities described in a study of 48 MEC patients were as follows: *CDKN2A* (41.6%), *TP53* (39.6%), *CDKN2B* (29.2%), *BAP1* (20.8%), *PIK3CA* (20.8%), *HRAS* (10.4%), *BRCA* (10.5%) mutations, and *ERBB2* amplifications (8.3%) (16). The latter, though infrequent, may be amenable to Her-2 targeted therapy (17). *TP53* mutation is one of the most common genomic alterations in MEC and is associated with the transformation of low-grade into high-grade tumors (12). In high-grade MEC, EGFR is overexpressed in 72.7% and was associated with a more aggressive behavior (18).

## Salivary Duct Carcinoma

Salivary duct carcinoma (SDC) is an aggressive subtype of SGC that microscopically resembles high-grade ductal carcinoma of the breast. They can develop as *de novo* disease or arise from a pleomorphic adenoma (carcinoma ex-pleomorphic adenoma) (19). The first line of treatment is currently based on platinum chemotherapy, with low response rates and of short duration (9). Biomarkers of interest have been found within this subtype, showing promising results with targeted therapy.

Androgen receptor (AR) and Her-2 receptors are frequently overexpressed in SDC. In a study of 177 patients with SDC, AR was expressed in 96% of cases (20). Her-2 overexpression can be found in one third to two thirds of cases, by immunohistochemistry and/or fluorescent *in situ* hybridization (FISH) (20, 21). These markers were not associated with disease biology and prognosis.

As in breast cancer, patients with SDC, and Her-2 overexpression derive benefit from anti-Her-2 therapy. In a phase II study, 57 patients with advanced SDC received docetaxel and trastuzumab, with an objective response rate (ORR) of 70.2%. The median progression-free survival (PFS) was 8.9 months and overall survival (OS) was 39.7 months (22).

The use of double Her-2 blockade with trastuzumab and pertuzumab was also evaluated in a basket study, which included five patients with advanced, refractory SDC, all with Her-2 amplification/overexpression. Trastuzumab and pertuzumab, without chemotherapy, yielded a partial response in four out of five patients with Her-2-positive SDC (ORR of 80%) (23).

Ado-trastuzumab emtansine (T-DM1) was also studied in another basket trial, where 10 patients with a median of two previous systemic treatments and HER-2 amplification by next-generation sequencing (NGS) had an ORR of 90%, half of which were complete metabolic responses. Median duration of response and PFS had not been reached with a median follow-up of 12 months (24). In this same study, the amplification of HER-2 by NGS correlated well with HER2/CEP17 ≥2 by FISH or IHC 3+ (24).

Treatment with androgen deprivation therapy (ADT) has been proposed after progression to platinum-based chemotherapy when AR is present. In a phase II study, 36 patients with metastatic or locally advanced unresectable SGC, being 34 SDCs, received combined androgen blockade with the luteinizing hormone-releasing hormone (LHRH) analog leuprorelin associated with bicalutamide, with an ORR of 41.7%. The median PFS was 8.8 months and median OS was 30.5 months. The treatment was well-tolerated, with a low rate of toxicity (25). ADT was also studied in the adjuvant setting in a retrospective study. Stage IVA/B, AR-positive SDC patients who underwent a complete tumor resection received bicalutamide, an LHRH analog or a combination of both. The treatment was associated with a statistically significant increase in the 3-year disease-free survival when compared to a control group (48.2 vs. 27.7%) (26). A randomized phase II study comparing the efficacy and safety of ADT with platinum-based chemotherapy as first-line therapy for patients with metastatic SDC and AR expression is ongoing (NCT01969578).

Enzalutamide, a more selective AR inhibitor, was given as monotherapy to patients with AR-positive SGC in a phase II trial (27). The majority (85%) of patients had SDC and 32.6% had prior AR-directed therapy. This study showed that 7 out of 46 patients (15%) had a partial response as best response, but only 4% (2/46) maintained the response until 8 weeks, thus failing to meet its primary endpoint. Therefore, we favor the administration of an antiandrogen agent in combination with an LHRH analog.

The experience of patients with prostate cancer can again be used in patients with SDC. Mechanisms of AR blockade resistance have been discovered in castration-resistant prostate cancer patients. The AR isoform splice variant 7 (AR-V7) results in a truncated receptor that lacks the binding site for androgen, activated even in the absence of ligands and stimulating tumor growth. Detection of AR-V7 in circulating tumor cells from patients with castrate-resistant metastatic prostate cancer was associated with worse PFS and OS in patients who received abiraterone or enzalutamide (28). In salivary duct carcinomas, the prevalence of AR-V7 is high, varying between 48 and 70% (29, 30). Interestingly, it is frequently detected in treatment-naive patients, as opposed to a mechanism of resistance to ADT as in prostate cancer. Therefore, its role in ADT sensitivity in SDC patients remains to be established, warranting further biomarker analysis in future trials. One case report of a patient with AR-positive SDC who expressed AR-V7 did not show response to second-line hormonal therapy with abiraterone (31).

Other potentially targetable pathways found in 28 SDC patients include *TP53* (68%), *HRAS* (25%), *PIK3CA* (18%), *NF1* (18%), *PTEN* (10%), *BRAF* (7%), and *NOTCH1* (7%). In the same study, patients did not have common predictive biomarkers of response to immunotherapy: 82% were PD-L1 negative, 91% had a low tumor mutational burden, and no patients presented microsatellite instability (29). Tipifarnib, a potent inhibitor of farnesyltransferase, an enzyme required for downstream signaling in HRAS-mutated tumors, was evaluated in 12 patients with SGC, with 4 being SDC, none of whom achieved a response. A single patient with acinic cell carcinoma had a partial response lasting at least 14 months (32).

## Secretory Carcinoma

Secretory carcinoma (SC), formerly known as mammary analog secretory carcinoma (MASC), was first described by Skálóva et al. a decade ago (33). It shows morphological, genetic, and immunohistochemical similarities with breast secretory carcinoma (34). One of the main differential diagnoses is acinic cell carcinoma (AcCC), which typically contains a basophilic cytoplasm with periodic acid-Schiff-positive zymogen granules and a more diverse cytologic profile compared to SC (35). SC has several architectural patterns (microcystic, solid, tubular, and cribriform), an abundant and eosinophilic cytoplasm, uniform proliferation and positivity for vimetin, mammaglobin, and S-100 protein in immunohistochemistry (36). The presence of a chromosomal translocation, t_(12, 15)_, between the *ETV6* gene on chromosome 12 with *NTRK3* on chromosome 15, generates the fusion product *ETV6*–*NTRK3*. It can be detected with a high specificity by reverse-transcriptase polymerase chain reaction (RT-PCR), NGS, or FISH, being the gold standard methods for the diagnosis of this subtype (33, 34). Nuclear pattern of pan-Trk immunohistochemistry staining has a good sensitivity to detect an *ETV6–NTRK3* fusion, thus aiding in differentiating SC from AcCC. However, it may miss other less frequent *ETV6-X* fusions, only detected by FISH or RT-PCR (37).

SC is more commonly found in men (55%), with a mean age of 44 years and mostly arising in the parotid gland, followed by several head and neck locations, including the oral cavity, submandibular glands, soft palate, buccal mucosa, base of tongue, and lips (38). It usually presents with an indolent clinical course and a good prognosis (39). Though a few cases of SC with high-grade histology and aggressive behavior have been described in association with *ETV6-MET* and *ETV6-RET* fusions, it has not yet been possible to correlate these recently described fusions with an overall behavioral pattern and disease prognosis (40, 41).

An *NTRK* fusion provides an actionable target for this disease by the Trk inhibitors larotrectinib and entrectinib. The benefit of larotrectinib was demonstrated by a phase II study including 12 cases of SC, with an objective response in 10 cases and an ORR of 80% by investigator's assessment (42). Entrectinib's activity was demonstrated by an integrated analysis of three phase I and II clinical trials (ALKA-372-001, STARTRK-1, and STARTRK-2), with the presence of seven (13%) cases of SC, which demonstrated an objective response in six of the seven cases (86%) (43). Both drugs received a tissue-agnostic FDA approval for tumors harboring an NTRK fusion.

Mechanisms of acquired resistance to larotrectinib have been described with an on-target mutation in the drug-binding site (42, 44). Selitrectinib (LOXO-195), a second-generation Trk inhibitor, was designed to overcome the acquired resistance to the first-line treatment. A phase I/II trial is ongoing (NCT03215511) and has evaluated 29 patients so far, with an ORR of 34% (45).

## Adenocarcinoma, Not Otherwise Specified

Adenocarcinoma, not otherwise specified (NOS), presents as a particularly difficult diagnosis to establish. It is characterized by the presence of areas of glandular or ductal differentiation mixed with a variety of specific growth patterns (46). Therefore, it is an exclusion diagnosis. The literature is controversial regarding its incidence among SGCs, ranging from 5 to 25% (3, 47). They are highly malignant tumors, with an overall 15-year survival rate of 3%, associated with early development of distant metastases and limited treatment options (48). Since this entity can share some characteristics of other SGCs, it is important to test for actionable biomarkers, such as AR and HER-2. Despite at a lower prevalence, they may be present and predict responses to targeted therapy (26, 49).

## Immunotherapy in Non-Adenoid Cystic Carcinoma

SGCs seem particularly resistant to immune checkpoint inhibitors. However, they represent a rather heterogeneous group of diseases that may behave differently in regard to the immune system. Linxweiler et al. demonstrated a distinct behavioral pattern in the different subtypes of SGCs. SDC exhibited higher levels of immune infiltration, T-cell dysfunction, and higher mutational load, whereas ACC presented with an overall lower mutational burden and an immune-excluded environment (50). PD-L1 expression was found to be associated with inferior disease-free survival (51).

Clinically, the KEYNOTE-028 study, a phase Ib basket trial, treated 26 patients with PD-L1-positive SGC with pembrolizumab at 10 mg/kg every 2 weeks. The low rate of PD-L1 positivity (<30%) limited patient accrual in the screening phase. Patients had adenocarcinoma, NOS (38%), mucoepidermoid (12%), undifferentiated (8%), squamous cell (8%), and ACC (8%). Despite being a PD-L1-enriched cohort, the results were overall disappointing, with an ORR of 12%. There were only three partial responses (two in adenocarcinoma, NOS and one in a high-grade serous carcinoma). The median PFS was 4 months (95% CI: 2 to 5 months) and median OS was 13 months (95% CI: 6 months to not reached) (52).

Another programmed-death 1 (PD-1) inhibitor is being evaluated in an ongoing phase II trial (NISCAHN trial). The use of nivolumab in 52 non-ACC patients demonstrated a 6-month non-progression rate (NPR_6M_) in 7 patients (14%, 90% CI: 6.8–24.7), with 2 partial responses (3.8%) and 22 patients with stable disease (42.3%). The median PFS was only 1.8 months (95% CI: 1.7–3.5) (53).

The role of tumor mutation burden (TMB) is unclear in SGCs. The subgroup analysis by TMB from the KEYNOTE-158 trial led to the approval of pembrolizumab for patients with TMB >10 mut/Mb as an agnostic treatment. There were three patients with salivary histologies and high TMB, one of whom achieved a partial response (54).

**Figure 1:**
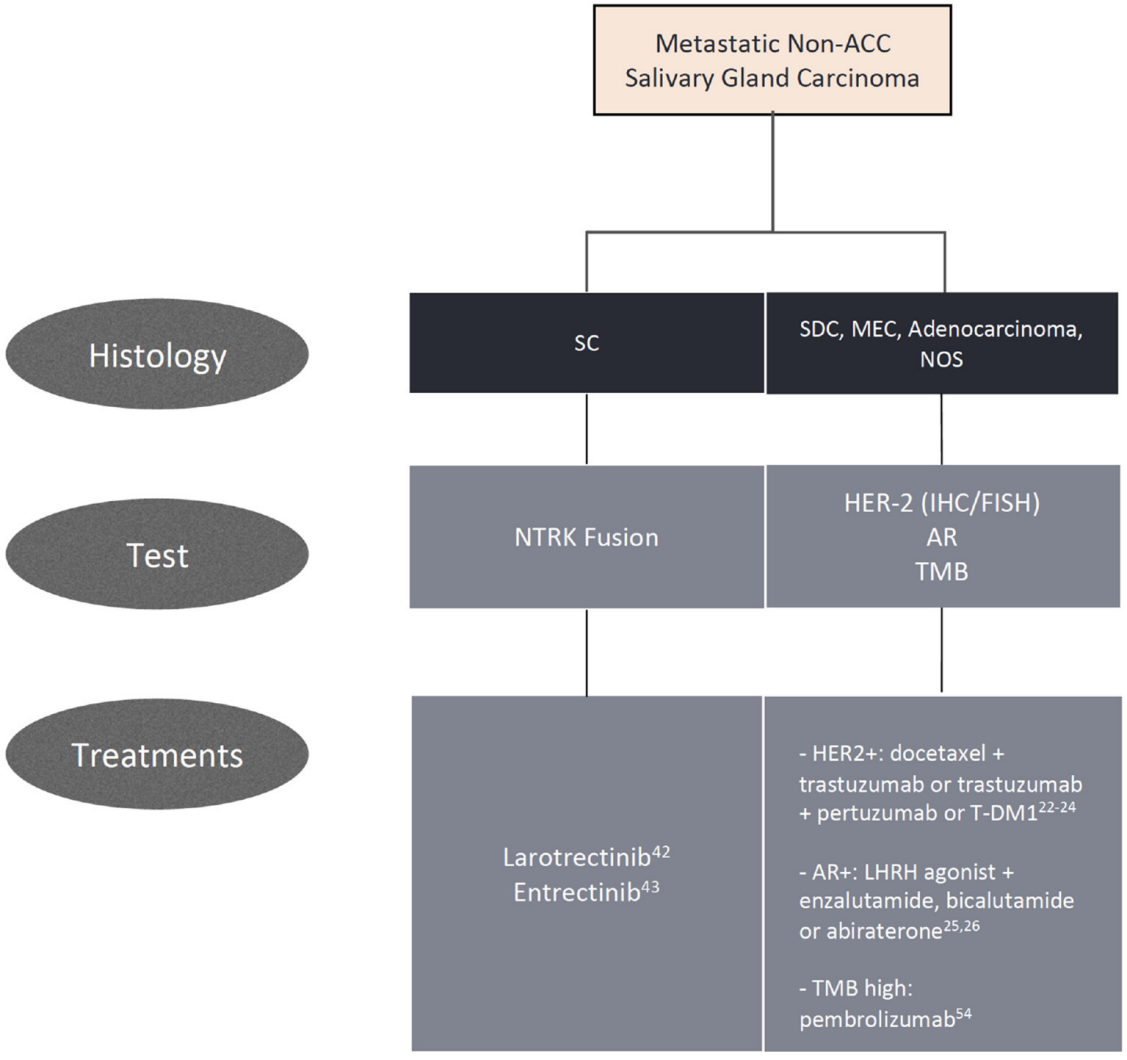
Algorithm for biomarker testing and treatment options in non-adenoid cystic carcinomas.

The addition of vorinostat, a histone deacetylase (HDAC) inhibitor, to pembrolizumab was evaluated in a phase I/II trial with 25 SGC patients. The association yielded a partial response in 4 patients (16%) and stable disease in 14 (56%), with a median PFS of 6.9 months and a median OS of 14 months (55). The combination of nivolumab and ipilimumab is being evaluated in an ongoing phase II study (NCT02834013). A summary of all relevant trials in non-ACC histologies is displayed in Table 1, and ongoing studies are shown in Table 2. We acknowledge the challenge in treating advanced SGC and propose practical alternatives to chemotherapy based on biomarkers in daily practice, displayed in Figure 1.

**Table 1:** All available data about advanced non-ACC therapy.

**Subtype**	**Study type**	**Drug**	**Number of patients**	**ORR (%)**	**mPFS, months**	**References**
All histologies	Phase II	Trastuzumab	10	0	4.2	(56)
All histologies	Phase IIa	Trastuzumab + pertuzumab	5	1	N/A	(23)
All histologies	Phase II	T-DM1	10	0.9	NR	(24)
All histologies	Phase II	T-DM1	3	0.7	N/A	(57)
All histologies	Phase II	Lapatinib	17	0	2.1	(58)
All histologies	Phase II	Enzalutamide	46	0	5.5	(27)
All histologies	Phase Ib	Pembrolizumab	26	0.1	4	(52)
All histologies	Phase II	Nivolumab	52	9	1.8	(53)
All histologies	Phase I/II	Pembrolizumab + vorinostat	25	0.2	7	(55)
All histologies	Phase II	Cetuximab	7	0	3.0	(59)
All histologies	Phase II	Gefitinib	18	0	2.1	(60)
All histologies	Phase II	Axitinib	20	0	5.5	(61)
All histologies	Phase II	Sorafenib	19	0.2	4.2	(62)
All histologies	Phase II	Pazopanib	20	0	6.7	(63)
All histologies	Phase II	Nintedanib	7	0	N/A	(64)
All histologies	Phase II	Dasatinib	14	0	N/A	(65)
Secretory carcinoma	Phase I/II	Larotrectinib	12	1	NR	(42)
Secretory carcinoma	Phase I/II	Entrectinib	7	1	11	(43)
Salivary duct carcinoma	Phase II	Trastuzumab + docetaxel	67	0.7	8.9	(22)
Salivary duct carcinoma	Retrospective study	Trastuzumab + paclitaxel + carboplatin	13	0.6	N/A	(66)
Salivary duct carcinoma	Retrospective study	Bicalutamide or bicalutamide + goserelin	35	0,18	4	(20)
Salivary duct carcinoma & NOS	Phase II	Leuprorelin acetate + bicalutamide	36	0.4	8.8	(25)

*ORR, overall response rate; mPFS, median progression free survival; N/A, not available; NR, not reached; NOS, adenocarcinoma; not otherwise specified*.

**Table 2:** Clinical ongoing trials in different types of non-ACC.

**Subtype**	**Target**	**Drug**	**Study type**	**Status**	**ClinicalTrials identifier**
All histologies	c-MET	Cabozantinib	Phase II	Active, not recruiting	NCT03729297
All histologies	PD-1	Nivolumab	Phase II	Active, not recruiting	NCT03132038
All histologies	PD-1 CTLA-4	Nivolumab + ipilimumab	Phase II	Active, not recruiting	NCT03146650
All histologies	PD-1	Pembrolizumab	Phase II	Recruiting	NCT02628067
All histologies	PD-1 CTLA-4	Nivolumab + ipilimumab	Phase II	Recruiting	NCT02834013
All histologies	AR PD-1	Goserelin + pembrolizumab	Phase II	Recruiting	NCT03942653
All histologies	AR	Abiraterone	Phase II	Recruiting	NCT02867852
All histologies	AR	Apalutamide + GnRH	Phase II	Recruiting	NCT04325828
All histologies	NTRK	Selitrectinib	Phase II	Recruiting	NCT03215511
All histologies	MDM2	APG-115	Phase I/II	Recruiting	NCT03781986
All histologies	PD-1 VEGF	Pembrolizumab + lenvatinib	Phase II	Not yet recruiting	NCT04209660
Salivary duct carcinoma & NOS	AR	Bicalutamide + triptorelin	Randomized Phase II	Recruiting	NCT01969578

*GnRH, gonadotropin-releasing hormone; AR, androgen receptor*.

## Adenoid Cystic Carcinoma

ACC is the second most common malignant salivary neoplasm, accounting for around one quarter of cases. It is more frequently diagnosed in females, affecting all age groups and often arising from the minor salivary glands (3, 67).

ACC usually has an indolent course, albeit difficult to eradicate due to its persistent nature and recurrent growth pattern, with predilection for perineural invasion. The literature demonstrates that 5-year disease-free survival in patients with ACC is only 30–40% (67). ACC commonly metastasizes to lungs, bones, and liver, with a median OS of 20–32 months in this setting (68).

**Figure 2:**
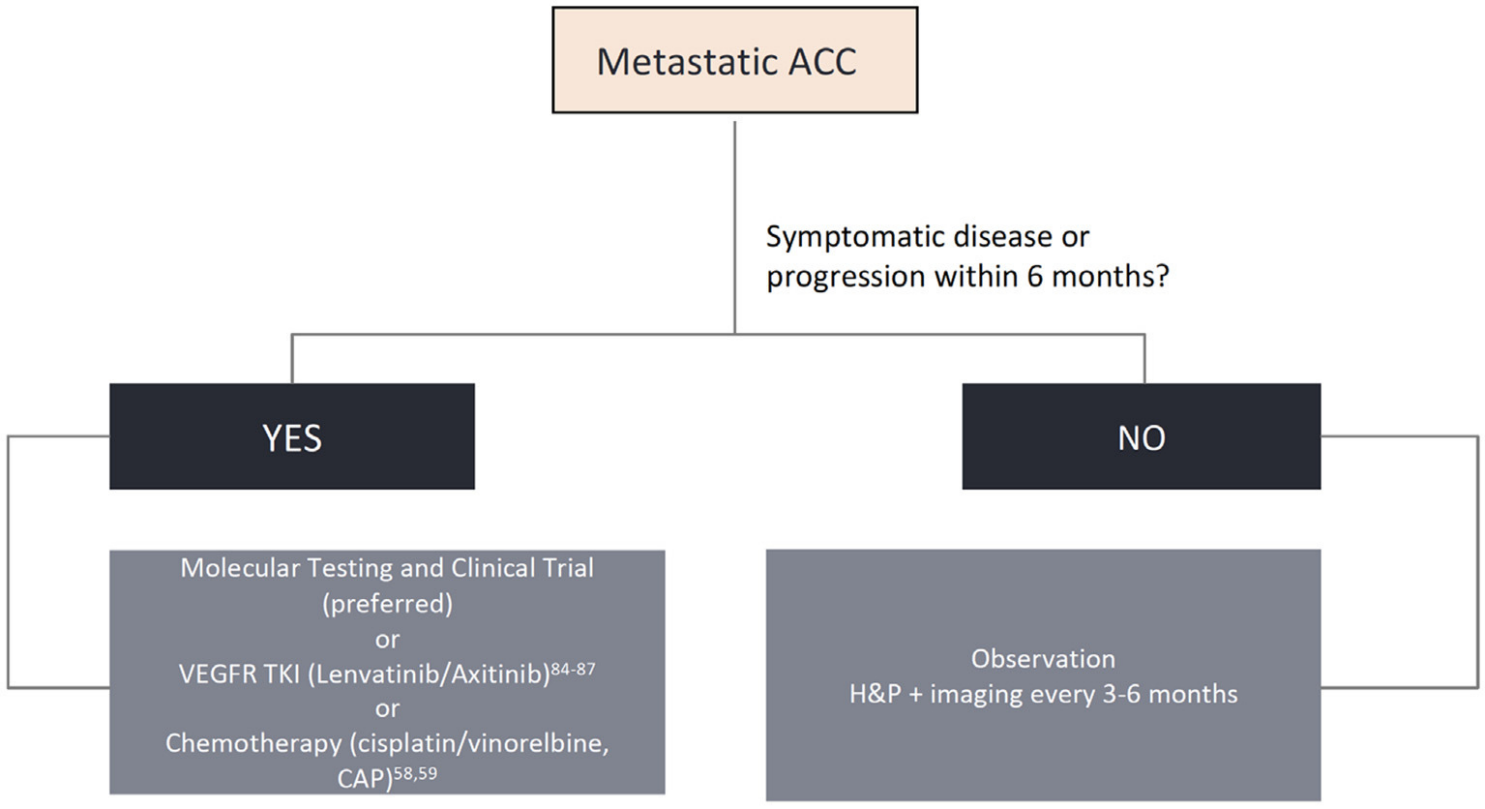
Algorithm for biomarker testing and treatment options in adenoid cystic carcinomas.

While surgery, with or without postoperative radiotherapy, is the mainstay treatment for localized disease, systemic therapy is reserved to the metastatic or unresectable locally advanced setting, with poor response rates and no consensus about the proper timing to be initiated. In this section, we will review proliferation pathways, molecular insights, and the development of new targeted drugs for patients with advanced disease. Though several actionable pathways are under scrutiny, limited evidence can aid in clinical practice. We propose a practical approach for newly diagnosed advanced ACC and options for later lines of therapy in Figure 2. Ongoing clinical trials are displayed in Table 3 and a summary of the main ACC studies conducted to date are displayed in Table 4.

**Table 3:** Clinical ongoing trials in ACC.

**Subtype**	**Target**	**Drug**	**Study type**	**Status**	**ClinicalTrials identifier**
All histologies	c-MET	Cabozantinib	Phase II	Active, not recruiting	NCT03729297
All histologies	PD-1	Nivolumab	Phase II	Active, not recruiting	NCT03132038
All histologies	PD-1 CTLA-4	Nivolumab + ipilimumab	Phase II	Active, not recruiting	NCT03146650
All histologies	PD-1	Pembrolizumab	Phase II	Recruiting	NCT02628067
All histologies	PD-1 CTLA-4	Nivolumab + ipilimumab	Phase II	Recruiting	NCT02834013
All histologies	PD-1 VEGFR	Pembrolizumab + lenvatinib	Phase II	Not yet recruiting	NCT04209660
All histologies	PSMA	Lutetium-177 PSMA	Phase II	Not yet recruiting	NCT04291300
Adenoid cystic only	VEGFR PD-L1	Axitinib + avelumab	Phase II	Recruiting	NCT03990571
Adenoid cystic carcinoma + other tumors	NOTCH	CB-103	Phase I/II	Recruiting	NCT03422679
All histologies + other tumors	NOTCH	BBI503 (amcarsetinib)	Phase Ib/II	Active, not recruiting	NCT01781455
Adenoid cystic only	NOTCH	AL 101	Phase II	Recruiting	NCT03691207
Solid tumors	MYB	TeTMYB + BGBA17	Phase I	Not yet recruiting	NCT03287427

**Table 4:** Available data about advanced ACC therapy.

**Subtype**	**Study type**	**Drug**	**Number of patients**	**ORR (%)**	**mPFS, months**	**References**
ACC	Phase Ib	Pembrolizumab	2	0	4	(52)
ACC	Phase I/II	Pembrolizumab + vorinostat	12	0	7	(55)
ACC	Phase II	Cetuximab	23	0	3.0	(59)
ACC	Phase II	Gefitinib	18	0	4.3	(60)
ACC	Phase II	Sorafenib	19	0	11.3	(69)
ACC	Phase II	Sorafenib	19	0	8.9	(62)
ACC	Phase II	Axitinib	33	0	5.7	(70)
ACC	Phase II	Regorafenib	38	0	N/A	(71)
ACC	Phase II	Lenvatinib	28	0	9.0	(72)
ACC	Phase II	Lenvatinib	32	0.2	17.5	(73)
ACC	Phase II	Nintedanib	65	0	8.2	(64)
ACC	Phase II	Dovitinib	34	0	8.2	(74)
ACC	Phase II	Imatinib	16	0	N/A	(75)
ACC	Phase II	Imatinib	17	0	N/A	(76)
ACC	Phase II	Dasatinib	40	0	4.8	(65)
ACC	Phase II	Lapatinib	33	0	3.5	(58)
ACC	Phase II	Everolimus	34	0	11.2	(77)
ACC	Phase II	Bortezomib + doxorrubicina	24	0,04	6.4	(78)
ACC	Phase I	GSK3326595^**^	14	0.2	N/A	(79)
ACC	Phase II	Vorinostat	30	0.7	7,7	(80)
ACC	Phase I	Bronticizumab	12	0.2	N/A	(81)
ACC	Phase Ib/II	Amcasertib (BBI503)^**^	14	0	6.1	(82)
ACC	Phase I	AL101	2	0	8	(83)
ACC	Phase I	Crenigacestat (LY3039478)	22	0	5.3	(84)

** *Trials ongoing with preliminary results*.

## Chemotherapy

Despite response rates of <30%, chemotherapy remains one of the most used treatments for this condition (85). The most consolidated regimen consists of cisplatin, doxorubicin and cyclophosphamide (CAP) (86). The best time to start treatment remains controversial, though it is commonly deferred until either symptomatic disease or a more accelerated growth pattern. Other cytotoxic agents have also been shown to be minimally active, such as mitoxantrone and vinorelbine, though other drugs such as paclitaxel should be avoided as single agents due to lack of proven efficacy (85).

## MYB–NFIB Pathway

Myb, a nuclear transcription factor, is overexpressed in 60–80% of ACCs, usually correlated with a genetic translocation of the *MYB* gene to the transcription factor gene *NFIB*, resulting in the *MYB-NFIB* fusion, an important oncogene (t_[6, 9]_). This fusion has been postulated as the main driver of tumor proliferation in ACC (87, 88). The Myb protein has an N-terminal DNA-binding domain and a central transactivation domain that regulate genes involved in cell cycle control, such as *NSR, MET, EGFR, IGF1R*, and specifically *IGF2* (89). The latter, by autocrine stimulation, controls the expression of the *MYB-NFIB* fusion in ACC cells, increasing proliferation and generating changes in the cell cycle and RNA processing (89–91). Other *MYB*-related fusions were described, however at lower frequencies than *MYB-NIFB*. Myb overexpression can also occur in the absence of detectable genetic alterations, implying that unknown pathways may be involved in its expression at the protein level (89).

Pre-clinical studies evaluated the role of targeted therapies, such as linsitinib (Igf1r inhibitor), gefitinib (EGFR inhibitor), and crizotinib (Alk and Met inhibitor) *in vitro* both as monotherapies and as a triplet regimen. Individually, none showed encouraging results, whereas a significant reduction of Myb expression was seen with the triplet regimen, suggesting a potential clinical benefit (92). *In vivo* studies are necessary to confirm activity in clinical practice with a tolerable toxicity profile, a major concern of combining these drugs.

More recently, the use of transretinoic acid (ATRA) showed interesting results in pre-clinical models. The drug reduced Myb binding in intensifying regions in *MYB*-translocated patient-derived xenograft models, thereby reducing the positive feedback for Myb overexpression cycle and thus reducing tumor proliferation (93). Two clinical trials are underway to address its role in treating patients with advanced ACC (NCT03999684; NCT04433169). Additionally, a study evaluating a Myb vaccine in combination with a novel anti-PD-1 is being conducted (NCT03287427).

## NOTCH1, 2, 3

Notch are transmembrane proteins that bind to neighboring cells and activate a biochemical cascade that gives rise to the process of cell differentiation, in addition to acting in the process of lateral regulation, proliferation, and angiogenesis of cells through the MAPK pathways (87). Mutations in the *NOTCH* gene family, particularly *NOTCH1*, are present in around 20% of ACC patients and are potential oncogenic drivers. The presence of this mutation characterizes a population with more advanced disease, along with the presence of bone and liver metastases and worse outcomes compared to a wild-type population (94).

A phase I trial tested the efficacy of brontictuzumab (OMP-52M51), a humanized monoclonal antibody against the Notch1 protein in a basket trial for solid tumors. Twelve patients (25%) had a diagnosis of ACC, with two developing a partial response and three with stable disease as best response, with tolerable adverse events (81). Another phase Ib/II study is evaluating the role of amcasertib (BBI-553), a cancer stemness kinase inhibitor that impairs cancer stem cell survival, which is intimately related to deregulated Notch pathway activity (95). Preliminary results demonstrated a disease control rate of 86% and median overall survival of 28.3 months (82). AL101, a γ-secretase inhibitor, also works by inhibiting the Notch pathway during the cleavage process for Notch's protein action in the intracellular domain. A phase I basket trial revealed a partial response lasting 8 months in 1 of 2 patients with ACC accrued (83). The phase II trial ACCURACY (NCT03691207) for ACC patients bearing *NOTCH* activating mutations is ongoing. A trial with another Notch inhibitor, CB103, is also being conducted (NCT03422679).

## Immunotherapy in ACC

The ACC cohort of the aforementioned KEYNOTE-028 represented only 8% of patients (*N* = 2), with none achieving a response. In terms of PFS and OS, results were poorer than with chemo or targeted therapy (52). Similarly, the combination of pembrolizumab in association with vorinostat was also disappointing in treatment of salivary gland tumors, including ACC, with low response rates (55). Nivolumab as a single agent was also evaluated in SGCs. In the ACC cohort, an ORR of 8.7% was observed (4/46 patients) (53). The combination of ipilimumab and nivolumab was initially thought to improve outcomes; however, only 2 out of 32 patients treated achieved a partial response, with a median PFS of 19.3 weeks in a prospective study (96). As previously stated, ACC appears to lack immune infiltration and harbors a lower mutation burden, being unlikely to benefit from immunotherapy (50).

## EGFR Pathway

EGFR is commonly overexpressed in ACC, though its presence in normal salivary gland tissue precludes any conclusions in its role in cancer development. Mutations in genes related to the EGFR pathway, including *EGFR, RAS* family, *PIK3CA, BRAF*, and *AKT1* are also present in ACC (97). Activating mutations in *EGFR* can be found in 10% of cases, though unlikely to be driver oncogenes in this setting (98). A phase I study tested gefitinib at 250 mg/day in 18 patients with ACC, and no responses were observed (60). Cetuximab was also evaluated in a single-arm, phase II study of EGFR-overexpressing patients, with disappointing results (59). Lapatinib has also been studied in patients who showed overexpression of EGFR and/or Her-2, again with unremarkable outcomes. Clinical benefit with stable disease was achieved in 36% of patients, with no objective responses (58).

## PRMT-5

PRMT5 is an enzyme that methylates arginines in proteins important for tumor growth and development (99). The phase I basket trial METEOR-1 evaluated the role of GSK3326595, a potent and selective PRMT5 inhibitor. Of the selected patients, 14 (26%) had metastatic ACC. Clinical activity was observed in several tumor types, notably with partial responses observed in 3/14 ACC cases, with tolerable adverse events (79).

## Histone Deacetylation

Epigenetic changes were found in most studies that carried out NGS. The acetylation of histone pathways, with mutations in chromatin remodeling genes, such as *SMARCA2, CREBBP*, and *KDM6A*, suggests aberrant epigenetic regulation in ACC oncogenesis (100). A pre-clinical study combining cisplatin and vorinostat found a remarkable efficacy in depleting CSCs and reducing tumor viability in all ACC primary cells (101). A phase II trial of vorinostat in ACC showed a partial response in 2/30 patients and stable disease in another 27 patients (80). However, a phase II trial combining vorinostat and pembrolizumab for recurrent or metastatic salivary gland cancer, as aforementioned, showed disappointing results, likely reflecting the immune-tolerant environment of ACC (55).

## KIT/VEGFR

Other overexpressed potential target receptors in ACC are the vascular endothelial growth factor receptor (VEGFR) and fibroblast growth factor receptor 1 (FGFR1). These are well-established oncogenic pathways and can be inhibited by anti-VEGFR/FGFR drugs (102). Sorafenib, nintedanib, axitinib, regorafenib, dovitinib, and other multi-kinase inhibitors were tested and showed only a modest benefit, with few objective response rates (Table 4). Notably, lenvatinib was evaluated in a population with metastatic ACC, who had already received up to one line of chemotherapy. A total of 28 patients were enrolled in the study, and 11.5% showed a partial response (72). Additionally, 25 to 27% of patients with ACC had at least 20% reduction in target lesion size. The median PFS and OS were 9.1 and 27 months, respectively. Despite the encouraging results, 50% of the patients presented grade 3 toxicity and dose reductions were necessary in most of the study population. Similarly, Tchekmedyian et al. conducted another phase II study with lenvatinib, with a 15.6% ORR and a remarkable median PFS of 17.2 months (73). Axitinib is another multi-kinase inhibitor with interesting results in ACC, but with a lower ORR and median PFS (9.1% and 5.7 months, respectively) (70). More recently, the first randomized phase II trial of its kind showed a significant improvement in PFS with axitinib vs. observation (HR: 0.25; 95% CI: 0.14–0.42; *P* < 0.0001), but with no improvement in OS (HR: 0.6; 95% CI: 0.26–1.38; *P* = 0.23) (103). In this study, none of the 27 patients treated achieved a response, but all (100%) had stable disease. This rekindles the discussion of whether deferring treatment until a more symptomatic or aggressive course of disease remains acceptable. We favor the use of lenvatinib due to its numerical superiority in ORR and PFS compared to axitinib, but starting at a lower dose of 20 mg/day, with subsequent dose escalation if adequately tolerated.

Despite the high percentages (90%) of overexpression of c-Kit by IHC in ACC, targeted agents such as imatinib and dasatinib failed to show a meaningful activity in this disease (65, 75, 76, 104). The best response was stable disease in 50% of the patients treated with dasatinib (65). The disappointing outcomes likely result from the lack of an underlying gene amplification and/or a *KIT* activating mutation, such as seen in other malignancies (gastrointestinal stromal tumors and chronic myeloid leukemia).

## ^177^Lu-PSMA

ACC cells can express prostate-specific membrane antigen (PSMA) in over 90% of cases, with significant uptake in PSMA-PET/CT (105). Such as in prostate cancer, this can be useful not only for staging and surveillance but also as an opportunity for PSMA-directed therapy. Lutetium-177 (^177^Lu)-PSMA is a radiolabeled small molecule that binds with high affinity to PSMA, enabling beta particle therapy targeted to metastatic castration-resistant prostate cancer, with promising results in this tumor type (106). A single case report so far has been reported in ACC, with a transient pain relief after one dose. However, the patient died within 6 weeks due to a highly refractory and advanced tumor (107). An ongoing clinical trial is prospectively evaluating the role of ^177^Lu-PSMA in advanced ACC (NCT04291300).

## Conclusions

In conclusion, SGCs may be challenging to treat due to its several histological subtypes. Molecular diagnostics are able to aid in diagnosis and guide discovery for subtype-specific targeted therapy. Currently, significant efforts are being undertaken to improve outcomes for advanced disease with biomarker-driven research. Given the limited efficacy with chemotherapy, a more personalized approach is of utmost importance to move forward in the management of this infrequent entity.

## Author Contributions

LD, IS, FT, RF, and GS participated in the concept design, writing, review, and approval of the final manuscript. All authors contributed to the article and approved the submitted version.

## Conflict of Interest

The authors declare that the research was conducted in the absence of any commercial or financial relationships that could be construed as a potential conflict of interest.
