# Corrigendum: Salivary Gland Carcinoma: Novel Targets to Overcome Treatment Resistance in Advanced Disease

**DOI:** 10.3389/fonc.2021.669486

**Published:** 2021-04-09

**Authors:** Larissa Di Villeneuve, Ive Lima Souza, Fernanda Davila Sampaio Tolentino, Renata Ferrarotto, Gustavo Schvartsman

**Affiliations:** ^1^ Department of Medical Oncology, Hospital Israelita Albert Einstein, São Paulo, Brazil; ^2^ Department of Head and Neck Medical Oncology, The University of Texas MD Anderson Cancer Center, Houston, TX, United States

**Keywords:** salivary gland cancer, molecular targeted therapy, androgen receptor, immunotherapy, ERBB-2 receptor, gene fusion, drug resistance

In the original article, there was a mistake in ***Figure 1****and****Figure 2*** as published. **The proposed algorithms were in disagreement among all authors and thus corrected.** The corrected *Figures* appears below.

**Figure 1:**
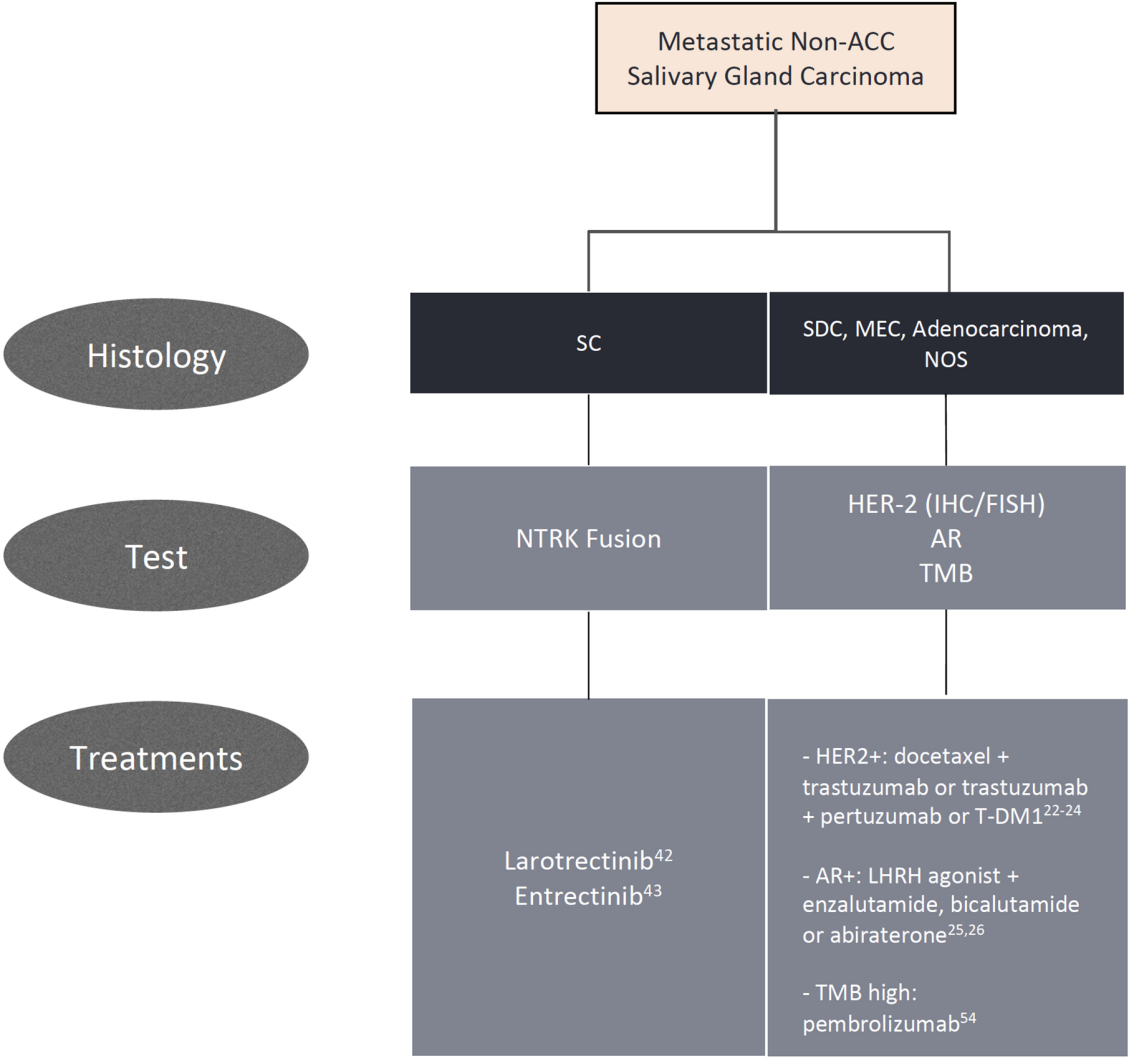
Algorithm for biomarker testing and treatment options in non-adenoid cystic carcinomas.

**Figure 2:**
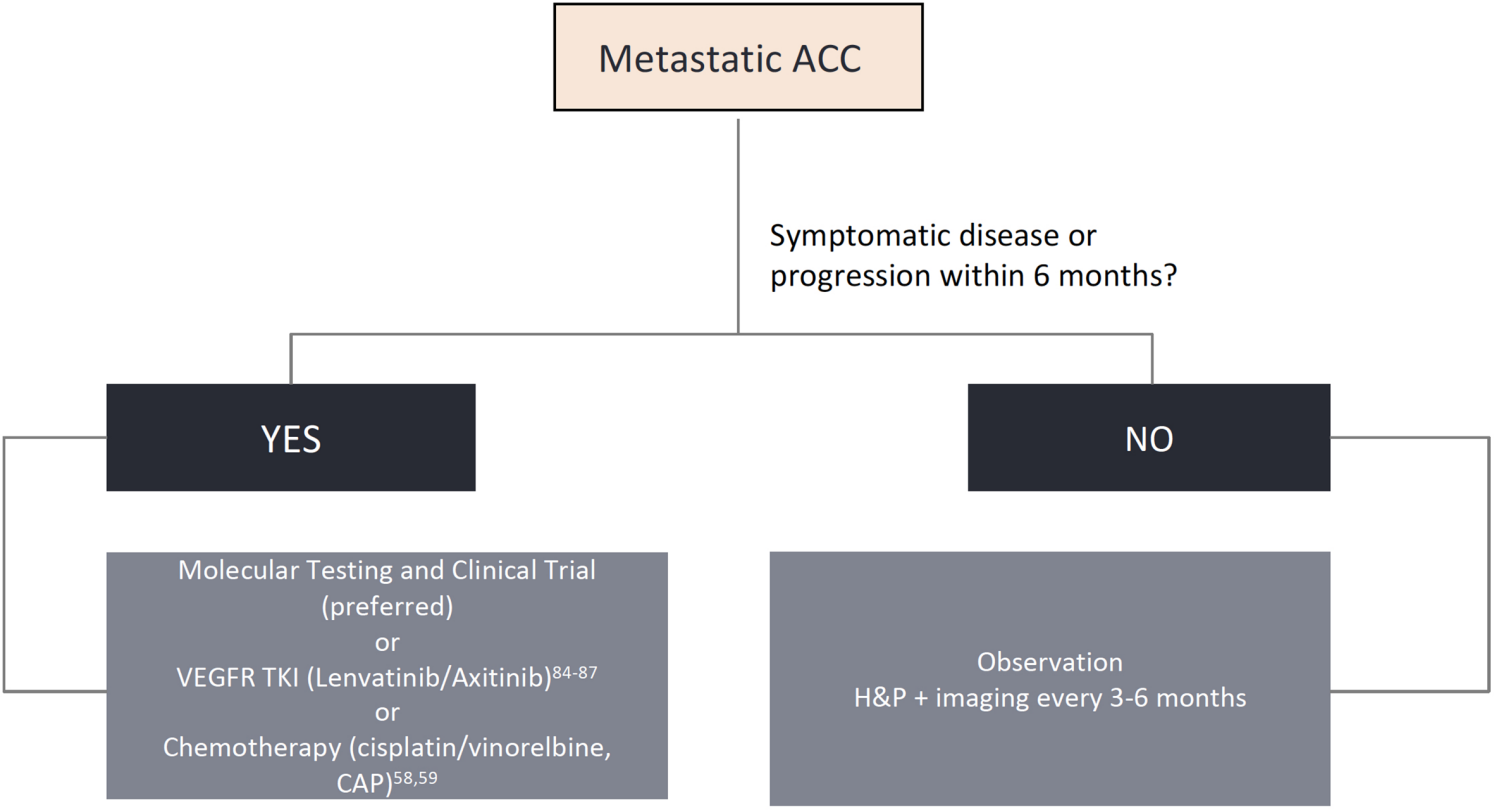
Algorithm for biomarker testing and treatment options in adenoid cystic carcinomas.

In the original article, there was an error. Myb protein was stated as a surface protein, which is incorrect.

Corrections have been made to:

1. Abstract:

Salivary gland carcinomas (SGC) account for less than 5% of head and neck malignant neoplasms, further subcategorized in over 20 histological subtypes. For the most part, treatment for advanced disease is guided by morphology. SGC in general respond poorly to standard chemotherapy, with short durability and significant toxicity. More recently, next-generation sequencing provided significant input on the molecular characterization of each SGC subtype, not only improving diagnostic differentiation between morphologically similar tumor types, but also identifying novel driver pathways that determine tumor biology and may be amenable to targeted therapy. Amongst the most common histological subtype is adenoid cystic carcinoma, which often harbors a chromosome translocation resulting in a MYB-NFIB oncogene, with various degrees of Myb expression. In a smaller subset, NOTCH1 mutations occur, conferring a more aggressive disease and potential sensitivity to Notch inhibitors. Salivary duct carcinomas may overexpress Her-2 and androgen receptor, with promising clinical outcomes after exposure to targeted therapies approved for other indications. Secretory carcinoma, previously known as mammary analogue secretory carcinoma, is distinguished by an ETV6-NTRK3 fusion that can both help differentiate it from its morphologically similar acinar cell carcinoma and also make it susceptible to Trk inhibitors. In the present article, we discuss the molecular abnormalities, their impact on tumor biology, and therapeutic opportunities for the most common SGC subtypes and review published and ongoing clinical trials and future perspectives for this rare diseases.

2. Section “MYB-NFIB pathway”, first paragraph:

Myb, a nuclear transcription factor, is overexpressed in 60-80% of ACCs, usually correlated with a genetic translocation of the MYB gene to the transcription factor gene NFIB, resulting in the MYB-NFIB fusion, an important oncogene (t(6;9)). This fusion has been postulated as the main driver of tumor proliferation in ACC (60,61). The Myb protein has an N-terminal DNA-binding domain and a central transactivation domain that regulate genes involved in cell cycle control, such as NSR, MET, EGFR, IGF1R, and specifically IGF2 (62). The latter, by autocrine stimulation, controls the expression of the MYB-NFIB fusion in ACC cells, increasing proliferation and generating changes in the cell cycle and RNA processing (62,63,64). Other MYB-related fusions were described, however at lower frequencies than MYB-NIFB. Myb overexpression can also occur in the absence of detectable genetic alterations, implying that unknown pathways may be involved in its expression at the protein level (62).

The authors apologize for these errors and state that these do not change the scientific conclusions of the article in any way. The original article has been updated.

## Conflict of Interest

The authors declare that the research was conducted in the absence of any commercial or financial relationships that could be construed as a potential conflict of interest.

